# Supplementary material for: EnzML: multi-label prediction of enzyme classes using InterPro signatures
Source: BMC Bioinformatics. 2012 Apr 25;13:61. doi: 10.1186/1471-2105-13-61 (PMC3483700; doi:10.1186/1471-2105-13-61)
Supplement: Addtional file 5 — The Java code to format the data files, evaluate and predict. The file enzml_java_code.tar.gz contains the Java code used to format database data to ARFF and XML formats, to execute cross and train-test (jackknife) evaluations and to record evaluation results to database. More information is included in the readme.txt file and the Javadoc files. The code can be used with a MySQL database. To use a different database software, other JDBC drivers might be required. [file 1471-2105-13-61-S5.gz › java_code/utils/doc/cern/colt/PersistentObject.html]

PersistentObject


---


|  |  |  |
| --- | --- | --- |
| **Overview** \| **Package** \| **Class** \| **Use** \| **Tree** \| **Deprecated** \| **Index** \| **Help** | |  |
| PREV CLASS   NEXT CLASS | **FRAMES**    **NO FRAMES**     **All Classes** |  |
| SUMMARY: NESTED \| FIELD \| CONSTR \| METHOD | DETAIL: FIELD \| CONSTR \| METHOD |  |


---


## cern.colt Class PersistentObject

```
java.lang.Object
  cern.colt.PersistentObject
```

**All Implemented Interfaces:**: java.io.Serializable, java.lang.Cloneable

**Direct Known Subclasses:**: AbstractDistribution, RandomElement

---

``` public abstract class PersistentObject extends java.lang.Object implements java.io.Serializable, java.lang.Cloneable ```

This empty class is the common root for all persistent capable classes. If
this class inherits from java.lang.Object then all subclasses are
serializable with the standard Java serialization mechanism. If this class
inherits from com.objy.db.app.ooObj then all subclasses are
*additionally* serializable with the Objectivity ODBMS persistance
mechanism. Thus, by modifying the inheritance of this class the entire tree
of subclasses can be switched to Objectivity compatibility (and back) with
minimum effort.

**See Also:**: Serialized Form

---

| **Method Summary** | |
| --- | --- |
| `java.lang.Object` | `clone()`             Returns a copy of the receiver. |

**Methods inherited from class java.lang.Object**

`equals, getClass, hashCode, notify, notifyAll, toString, wait, wait, wait`

**Method Detail**

### clone

```
public java.lang.Object clone()
```

:   Returns a copy of the receiver. This default implementation does not
    nothing except making the otherwise protected clone method
    public.

    :   **Overrides:**: `clone` in class `java.lang.Object`
    :   **Returns:**: a copy of the receiver.


---


|  |  |  |
| --- | --- | --- |
| **Overview** \| **Package** \| **Class** \| **Use** \| **Tree** \| **Deprecated** \| **Index** \| **Help** | |  |
| PREV CLASS   NEXT CLASS | **FRAMES**    **NO FRAMES**     **All Classes** |  |
| SUMMARY: NESTED \| FIELD \| CONSTR \| METHOD | DETAIL: FIELD \| CONSTR \| METHOD |  |


---
